# Reliability across content areas in progress tests assessing medical knowledge: a Brazilian cross-sectional study with implications for medical education assessments

**DOI:** 10.1590/1516-3180.2023.0291.R1.13052024

**Published:** 2024-07-15

**Authors:** Pedro Tadao Hamamoto, Miriam Hashimoto, Alba Regina de Abreu Lima, Leandro Arthur Diehl, Neide Tomimura Costa, Patrícia Moretti Rehder, Samira Yarak, Maria Cristina de Andrade, Maria de Lourdes Marmorato Botta Hafner, Zilda Maria Tosta Ribeiro, Júlio César Moriguti, Angélica Maria Bicudo

**Affiliations:** I Physician, Assistant Professor, Departament of Pediatrics, Faculdade de Medicina de Botucatu, Universidade Estadual Paulista (UNESP), Botucatu (SP), Brazil.; II Biologist, Assistant Professor, Departament of Molecular Biology, Faculdade de Medicina de São José do Rio Preto (FAMERP), São José do Rio Preto (SP), Brazil.; III Biologist, Assistant Professor, Departament of Molecular Biology, Faculdade de Medicina de São José do Rio Preto (FAMERP), São José do Rio Preto (SP), Brazil.; IV Physician, Assistant Professor, Departament of Internal Medicine, Universidade Estadual de Londrina (UEL), Londrina (PR), Brazil.; V Physician, Assistant Professor, Departament of Internal Medicine, Universidade Estadual de Londrina (UEL), Londrina (PR), Brazil.; VI Physician, Assistant Professor, Departament of Tocogynecology, Universidade Estadual de Campinas (UNICAMP), Campinas (SP), Brazil.; VII Physician, Assistant Professor, Departament of Dermatology, Universidade Federal de São Paulo (UNIFESP), São Paulo (SP), Brazil.; VIII Physician, Assistant Professor, Departament of Pediatrics, Universidade Federal de São Paulo (UNIFESP), São Paulo (SP), Brazil.; IX Physician, Assistant Professor, Academic Assessment Center, Faculdade de Medicina de Marília (FAMEMA), Marília (SP), Brazil.; X Physician, Assistant Professor, Academic Assessment Center, Faculdade de Medicina de Marília (FAMEMA), Marília (SP), Brazil.; XI Physician, Associate Professor, Departament of Internal Medicine, Universidade de São Paulo (USP), Ribeirão Preto (SP), Brazil.; XII Physician, Full Professor. Departamentof Pediatrics, Universidade Estadual de Campinas (UNICAMP), Campinas (SP), Brazil.

**Keywords:** Internal medicine., Education, medical., Educational measurement., Reliability., Progress test., Medical education assessments.

## Abstract

**BACKGROUND:**

Brazilian medical schools equitably divide their medical education assessments into five content areas: internal medicine, surgery, pediatrics, obstetrics and gynecology, and public health. However, this division does not follow international patterns and may threaten the examinations’ reliability and validity.

**OBJECTIVE:**

To assess the reliability indices of the content areas of serial, cross-institutional progress test examinations.

**DESIGN AND SETTINGS:**

This was an analytical, observational, and cross-sectional study conducted at nine public medical schools (mainly from the state of São Paulo) with progress test examinations conducted between 2017 and 2023.

**METHODS:**

The examinations covered the areas of basic sciences, internal medicine, surgery, pediatrics, obstetrics and gynecology, and public health. We calculated reliability indices using Cronbach’s α, which indicates the internal consistency of a test. We used simple linear regressions to analyze temporal trends.

**RESULTS:**

The results showed that the Cronbach’s α for basic sciences and internal medicine presented lower values, whereas gynecology, obstetrics, and public health presented higher values. After changes in the number of items and the exclusion of basic sciences as a separate content area, internal medicine ranked highest in 2023. Individually, all content areas except pediatrics remained stable over time.

**CONCLUSIONS:**

Maintaining an equitable division in assessment content may lead to suboptimal results in terms of assessment reliability, especially for internal medicine. Therefore, content sampling of medical knowledge for general assessments should be reappraised.

## INTRODUCTION

Traditionally, Brazilian medical schools have divided general medical education assessments into five content areas: internal medicine, surgery, pediatrics, obstetrics and gynecology, and public health. This division was the consequence of a resolution passed in 2000 by the National Medical Residency Commission,^1^ which defined the organization of the selection processes nationally. Despite the several modifications to the resolution in subsequent years, the equitable division of the number of items between the five content areas has remains untouched.

Therefore, all medical selection processes for general specialties (e.g., internal medicine, pediatrics, and general surgery) and direct-access specialties (e.g., anesthesiology, ophthalmology, and radiology) are legally obligated to use this division. Similarly, many undergraduate medical curricula follow this division, either for conducting assessments, such as inter-institutional progress testing,^2^ or for organizing clerkship rotations.^3^

However, this equitable division of item numbers does not conform to international patterns. In the Netherlands, progress testing uses a two-dimensional blueprint that includes elements from disciplines (e.g., surgery, dermatology, pediatrics, and physiology) and categories (e.g., the respiratory and musculoskeletal systems).^4^ The German Progress Test Medizin is blueprinted according to organ systems with different percentages for each (e.g., 11% for the cardiac system and 4.5% for skin).^5^ Similarly, Step 2 of the United States Medical Licensing Examination includes differential weighing of content areas (e.g., 8–10% for the cardiovascular system and 4–6% for pregnancy, childbirth, and puerperium).^6^

Furthermore, the numbers of items across different content areas have implications for examination reliability, which is the reproducibility of assessment outcomes over time or on specific occasions, that is, the consistency of measurements. Therefore, owing to a large component of random errors, the data resulting from low-reliability assessments may threaten the generalization and interpretation of the results.^7^

## OBJECTIVE

This study aimed to assess the reliability indices of the content areas of serial, cross-institutional progress test examinations conducted in the state of São Paulo between 2017 and 2023.

## METHODS

### Study design

We conducted an analytical, observational, cross-sectional study based on data from a retrospective database of inter-institutional progress test examinations held between 2017 and 2023 in nine public medical schools (mainly from the state of São Paulo). We considered only the grades achieved by sixth-year medical students as the test was designed at the level of a recently graduated physician. **Table 1** presents the number of participating students for each year.

**Table 1:** Number of participating sixth-year medical students across the observational period

Year	Number
2017	687
2018	742
2019	713
2020	712
2021	666
2022	726
2023	525
Total	4771

### Ethical considerations

We used only secondary data from the examinations and did not identify individual students; therefore, approval from an ethical review board was not necessary according to national legislation.

### Settings and progress test information

The participating schools joining a consortium were Universidade Estadual Paulista (UNESP), Universidade Estadual de Campinas (UNICAMP), Universidade de São Paulo (USP – Ribeirão Preto), USP – Bauru, Universidade Federal de São Paulo (UNIFESP), Universidade Federal de São Carlos (UFSCAR), Faculdade de Medicina de Marília (FAMEMA), Faculdade de Medicina de São José do Rio Preto (FAMERP), and Universidade Estadual de Londrina (UEL). Universidade Regional de Blumenau (FURB) participated in the progress test examinations from 2017 to 2022. Further, Universidade de São Paulo, Bauru campus was inaugurated in 2018; consequently, their sixth-year students participated in the progress test examinations only in 2023.

From 2017 to 2022, the progress test examination consisted of 120 multiple-choice questions equally divided into 20 items from basic sciences, internal medicine, pediatrics, surgery, gynecology and obstetrics, and public health. The use of basic sciences as a content area was related to the consortium’s specific history. However, in 2023, basic sciences-related content was distributed among the other five areas, and the number of items was changed to emphasize internal medicine more. Therefore, in 2023, the items were 34 for internal medicine, 23 for pediatrics, 23 for surgery, 20 for gynecology and obstetrics, and 20 for public health. The items were based on clinical vignettes and focused on applied knowledge rather than on the retrieval of memorized information.

### Data analysis

We calculated reliability indices using Cronbach’s α coefficients,^8^ which provide a measure of the internal consistency of a scale or test and range from 0 (low consistency) to 1 (high consistency). Tavakol and Dennick have stated that “*internal consistency describes the extent to which all the items in a test measure the same concept or construct and hence it is connected to the inter-relatedness of the items within the test.*”^9^ We calculated the α coefficients of each content area for each examination year using the following formula:


$$\alpha =\left(\frac{k}{k-1}\right)\;x\;(1-\frac{\sum_{i=1}^{k}s_{i}^{2}}{s_{sum}^{2}})$$

where *k* is the number of items in the test; $s_{1}^{2}$ is the variance of each item, and $s_{sum}^{2}$ is the variance in total scores for each respondent.

We tested the differences between the mean α coefficients using one-way analysis of variance (ANOVA) and calculated temporal trends in α coefficients using a simple linear regression model. The statistical significance level was set at P = 0.05. We performed statistical analyses using SPSS, version 24.0 (IBM Corp., Armonk, New York, United States) and Prism 9 for MacOS (version 9.5.0, GraphPad Software, San Diego, California, United States).

## RESULTS

**Figure 1:**
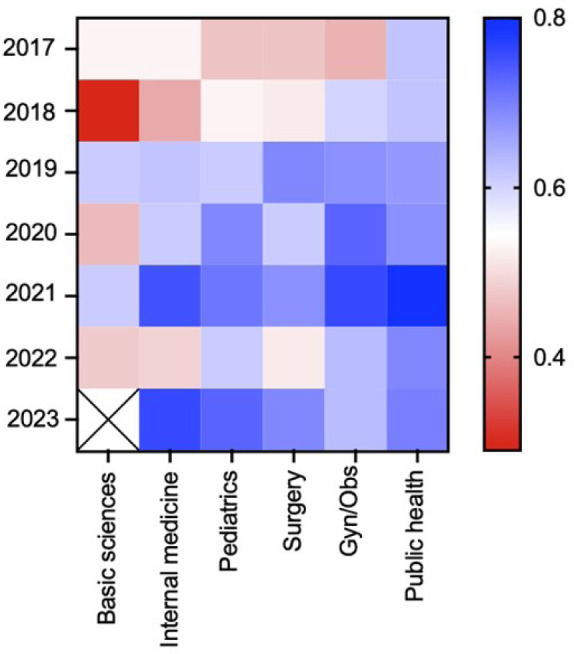
Heat map of alpha coefficient values for the six content areas between 2017 and 2023.

In terms of the absolute values of the α coefficients, basic sciences and internal medicine presented lower values, whereas gynecology and obstetrics, and public health presented higher values (**Figure 1**). After ranking the content areas according to a top-down classification of the α coefficients, we found that basic sciences ranked last in almost all years. Internal medicine had intermediate to low positions. Further, pediatrics and surgery had intermediate positions, while public health and gynecology and obstetrics were the highest-ranked areas. After the changes in the number of items and the absence of basic sciences as a separate content area in 2023, internal medicine had the highest position.

The mean α coefficients from 2017 to 2023 were 0.497, 0.600, 0.621, 0.597, 0.640, and 0.683 for basic sciences, internal medicine, pediatrics, surgery, gynecology and obstetrics, and public health, respectively. We found no statistically significant difference in the mean values (F = 2.432, P = 0.054).

**Figure 2:**
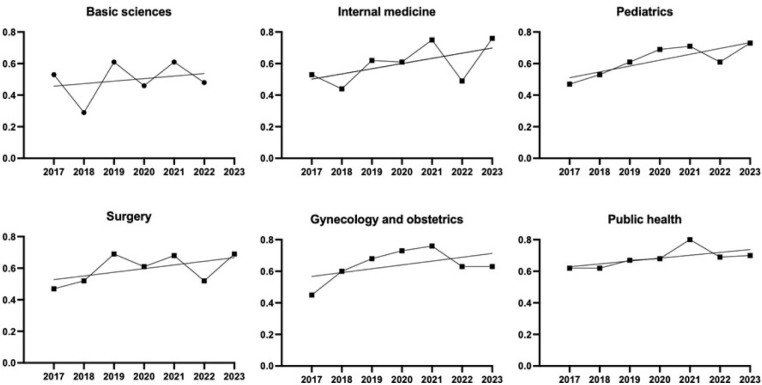
Alpha coefficient values (y axis) for each content area of progress testing between 2017 and 2023, with their respective trend lines. Basic sciences had the lowest intercept value and only pediatrics had a statistically significant upward trend.

**Figure 2** shows the analysis of the temporal trends of each area, which revealed a significant difference in the intercept values of the lines, with basic sciences and internal medicine presenting the lowest values (F = 2.897, P = 0.027). That is, the initial values of the trend lines were significantly lower in these two content areas. Individually, all content areas except pediatrics remained stable over time, and pediatrics presented a significant upward trend despite a slight decrease in 2022.

Regarding the slopes, that is, the annual change in trend line characteristics, we found no difference between the content areas (F = 0.206, P = 0.957). Therefore, despite the differences in the initial values, the lines remained parallel to each other, with a pooled slope of 0.026, which indicates a slight increase in the examinations’ overall consistency (**Table 2**).

**Table 2:** Linear regression models of temporal trends for each content area’s reliability index

Area	Intercept	Slope	R^2^	P value
Basic sciences	0.441	1.6%	0.063	0.632
Internal medicine	0.469	3.3%	0.332	0.176
Pediatrics	0.473	3.7%	0.691	**0.020**
Surgery	0.504	2.3%	0.288	0.214
Gynecology and obstetrics	0.543	2.4%	0.266	0.236
Public health	0.610	1.8%	0.420	0.116

The intercept refers to the point at which the regression line crosses the y axis. The slope refers to the annual percentage change. R^2^ is the determination coefficient: the closer to “1,” the better the adjustment of data to the regression. All areas showed a positive trend towards improvement in the reliability index, with a significant trend for Pediatrics (P < 0.05).

## DISCUSSION

The use of progress testing by medical schools is increasing in Brazil as feedback for students, faculty, and institutions effectively improves medical education.^2,10,11^

Created in the Netherlands and USA in the 1970s, progress testing began to be used in Brazil at Universidade de São Paulo and Universidade Estadual de Londrina between the late 1990s and early 2000s. The first progress testing examinations at Universidade de São Paulo consisted of 100–130 items divided into three areas: basic sciences, clinical sciences, and clerkship rotations. This division followed the traditional organization of medical curricula, and the number of items in each area was weighted according to the disciplines’ workload.^11^ At Universidade Estadual de Londrina, the test was divided into six areas: basic sciences, internal medicine, pediatrics, surgery, gynecology and obstetrics, and public health.^13^

This organization of curricula and assessments has been used in Brazil since the early 1990s owing to the immense efforts of the Interinstitutional Commission for the Evaluation of Medical Education (CINAEM).^14^ This commission has played an important role in restructuring medical education in Brazil, especially in assessing medical school processes.^15^ Their work culminated in the publication of the National Curricular Guidelines in 2001,^16^ which was a landmark for medical education settings. The guidelines were reviewed years later in 2014 to emphasize other important domains for recently graduated general physicians, such as mental health, urgency, and emergencies.^17,18^

However, the equitable division of the number of items in general medical education assessments has not yet been adequately reappraised. The present results demonstrate that following this distribution may lead to suboptimal results in terms of assessment reliability, especially for internal medicine. This is why the consortium of public medical schools in the state of São Paulo changed the number of items across the content areas in progress testing in 2023. Consequently, the reliability index for internal medicine, which ranked highest, immediately increased. The increase in the number of items alone may have led to this result as the α coefficient is considerably influenced by the number of items.^19^ While this may be true, it is only a part of the effect.

Following the well-accepted aphorism of biomedical research, increasing the sample size leads to more reliable results.^20^ Twenty items do not seem sufficient to adequately sample all the content that medical students should know about internal medicine. In addition, if internal consistency refers to the extent to which different items measure the same construct, it is worth imagining how distant an item about acute myocardial infarction may be from another item about osteoarthritis. In contrast, for obstetrics, an item addressing gestational hypertension is expected to be similar to an item referring to HELLP syndrome (i.e., these two items probably measure the same construct).

Incorporating basic sciences into the other content areas was a necessary adjustment to the test. As initially designed, progress tests must be structured at the graduate level, and therefore, writing items for “*first year students to get right*” is not adequate.^21^ Therefore, items testing knowledge about basic sciences should be framed at the functional level of a recently graduated physician. Increasing the number of items in all content areas would be ideal; however, the extent to which tiredness undermines students’ performance remains unclear. Therefore, we retained all 120 items.

We observed the best reliability indices for all six content areas in 2019, 2021, and 2023, and this is no coincidence. In those years, the progress test included pre-tested items, that is, items that had been previously used and selected based on their good psychometric properties. This finding highlights the benefits that medical schools can derive by supporting their faculty’s ability to write good items that obtain better assessments^22^; this is especially important for basic sciences, whose faculties are lesser experienced with writing clinical-based items.^23^ Good items have higher taxonomic levels and better psychometric behavior, and therefore, they provide more reliable results.^24,25^ Thus, better assessment is the key to improving medical education as it steers learning along the right path.^26^ Moreover, we observed that the α coefficients of all content areas has been increasing over the years, which indicates that this consortium of public medical schools in the state of São Paulo has improved the quality of the progress test examination.

Previous studies have highlighted the need to better explore the validity of assessments in medical education.^27,28^ In this context, validity measures the truth of the inferences made from the results of an examination. Reliability is not sufficient to guarantee validity but is a condition for it.^29^ Our study focused on a particular domain of validity—that is, a psychometric focus on reliability. Nevertheless, our results call attention to the importance of a broader perspective on medical education assessment and the need to reappraise content sampling of medical knowledge.
